# Chinese herbal medicines for the treatment of cough in idiopathic pulmonary fibrosis: A protocol for systematic review and meta-analysis

**DOI:** 10.1097/MD.0000000000022991

**Published:** 2020-10-30

**Authors:** Siyao Xiao, Yang Yu, Yimin Xiong, Fang Sun, Xiaoyu Liu, Jiaxin Yan, Shunan Zhang

**Affiliations:** a Graduate School, Beijing University of Chinese Medicine; b Department of Traditional Chinese Medicine for Pulmonary Diseases, Center of Respiratory Medicine, China-Japan Friendship Hospital, Beijing, China.

**Keywords:** Chinese herbal medicines, cough, idiopathic pulmonary fibrosis, protocol, systematic review

## Abstract

**Background:**

Idiopathic pulmonary fibrosis (IPF) is a chronic progressive disease with unknown etiology and hidden onset, which causes major health problems worldwide. Cough is a typical manifestation of IPF, which is usually characterized by cough without phlegm, and seriously affects the quality of life (QOL) of patients. At present, the treatment of IPF is mainly focused on prolonging survival time and improving lung function, such as pirfenidone, nintedanib, and N-acetylcysteine (NAC), but lack of effective measures to improve the QOL. Chinese herbal medicines (CHMs) is widely used in the clinical treatment of IPF. The adjuvant treatment of CHMs can effectively reduce the clinical symptoms of patients. Therefore, we designed this study to evaluate the role of CHMs in the treatment of cough in IPF.

**Method:**

This systematic review and meta-analysis will extract all randomized controlled trials (RCTs) related to the treatment of IPF from the following electronic database without date or language restrictions: PubMed, EMBASE, Cochrane CENTRAL, CNKI, VIP, CBM, and Wanfang database. The primary outcomes will be cough frequency and QOL, while secondary outcomes will include safety events. The methodologic quality of RCTs will be assessed using the Cochrane risk assessment tool. The I^2^ test will be used to identify the extent of heterogeneity, and funnel plot analysis will be used to test the publication deviation (the number of studies included >10). We will use RevMan5.3 software for data synthesis and analysis.

**Result:**

This review evaluates the efficacy and safety of CHMs in combination therapy on cough frequency, the quality of life, adverse reactions and safety incidents in patients with IPF.

**Conclusion:**

This study protocol will be used to evaluate the efficacy and safety of CHMs in combination with conventional therapy in treatment of cough in IPF.

**OSF Registration DOI:**

10.17605/OSF.IO/JKQYV.

## 1 Introduction

Idiopathic pulmonary fibrosis (IPF) is a chronic progressive fibrotic interstitial pneumonia of unknown etiology. IPF usually occurs in men aged 50 to 70 years old. Clinically, it is characterized by an insidious onset of breathlessness with exertion and a nonproductive cough, and the onset of the disease is hidden. IPF is a refractory lung disease with rapid progress and poor prognosis. The median survival time from respiratory symptoms to death is 2 to 3 years, and the 5-year survival rate is less than 30%.^[1,2]^ Cough is a common symptom of IPF, and it may also be a major symptom in some patients. Up to 80% of patients with IPF have a chronic cough. Cough caused by IPF is characterized by dry cough without sputum, which seriously affects the quality of life (QOL) of patients.^[3,4]^ Studies have shown that cough can predict the progression of the disease, even the first symptoms of many IPF patients, especially in patients with more severe pulmonary fibrosis. In IPF, the mechanism of cough is not fully understood. At present, it is believed that mechanical distortion of pulmonary parenchyma fibrosis, increased cough reflex sensitivity, gastroesophageal reflux, and airway inflammation may be potentially important mechanisms. In a CHEST guideline and expert panel report on cough associated interstitial lung disease (ILD) published in 2018, there are no effective drugs for the treatment of cough in IPF, so the treatment needs to be further explored at this stage.^[5]^ In the past meta-analysis of IPF, most studies focused on survival time and changes in lung function, but the assessment of cough and QOL is still blank.

The drugs commonly used in the treatment of IPF are NAC, pirfenidone, and nintedanib. Evidence of evidence-based medicine proves that NAC cannot improve the QOL of patients with IPF, nor can it reduce complications and mortality, and the therapeutic effect of IPF is controversial.^[6,7]^ Some clinical trials have shown that pirfenidone and nintedanib can reduce the decrease of forced expiratory vital capacity (forced vital capacity, FVC) and carbon monoxide diffusion capacity (carbon monoxide diffusing capacity, DLCO), and have protective effects on acute exacerbation and mortality, but do not affect the change of QOL. Only 1 small sample prospective observational study showed that pirfenidone could reduce the objective cough frequency, but the limitations of this trial were small sample size, limited inclusion inpatient criteria and short follow-up time.^[8–10]^ Two clinical trials on the treatment of IPF cough have found that thalidomide has a certain effect on IPF cough, but after weighing its efficacy and side effects, thalidomide is not recommended as a therapeutic drug.^[11,12]^ At present, there is no specific therapeutic drug for IPF. Traditional Chinese Medicine (TCM) has been used to treat cough in China for thousands of years, including chronic cough caused by pulmonary fibrosis. According to the consensus of Chinese experts on the diagnosis and treatment of IPF formulated in 2016, it is considered that TCM can be used to alleviate the symptoms of IPF patients according to the principle of dialectical treatment.^[13]^ Some evidence shows that TCM can improve the TCM syndrome of IPF patients and the SGRQ score indicating the QOL of patients, and there are few adverse events.^[14–16]^ However, in several existing systematic reviews and meta-analyses, the quality of RCTs included is generally not high. Most systematic reviews do not report changes in cough frequency and cough-related QOL, and there is a lack of high-quality evidence to prove the treatment of cough in IPF with TCM. Intending to fill the evidence gap, this is a systematic review and meta-analysis that will assess the effects and safety of the intervention, whether individual or combined, for the treatment of CHMs of the cough and QOL in patients with IPF.

## 2 Methods

This protocol is prospectively registered in the international prospective register of systematic reviews OSF Registration (OSF Registration DOI: 10.17605/OSF.IO/JKQYV) and the procedure is based on the Preferred Reporting Items for Systematic Review and Meta-Analysis Protocols (PRISMA-P) guidance.^[17,18]^ Besides, all research processes will follow the guidance of the Cochrane handbook.^[19]^

### 2.1 Inclusion criteria

#### 2.1.1 Types of studies

Only randomized controlled trials (RCTs) will be accepted. We will not include other types of clinical trials (e.g., case report, observational studies, retrospective studies, prospective studies) and animal trials in this study.

#### 2.1.2 Types of participants

The diagnostic criteria are based on the 2015 international evidence-based guidelines for IPF diagnosis and management jointly published by ATS/ERS/JRS/ALAT.^[1]^ In addition, we will also refer to the consensus of Chinese experts on the diagnosis and treatment of IPF formulated by the Interstitial Pulmonary Diseases Group of the Chinese Academy of Respiratory Medicine in 2016.^[13]^ In order to reduce the impact of other diseases on the treatment process and outcomes, patients with congenital heart disease, lung cancer, bronchiectasis, pneumonia, liver and kidney damage, and other systemic diseases will be excluded. The patients included regardless of age, severity, and duration of the diseases.

#### 2.1.3 Types of interventions

Interventions in the experimental group are CHMs or CHMs combine with conventional medicine treatment. The dosage form of CHMs are not limited, and Chinese medicine single prescription, Chinese medicine compound prescription, Chinese medicine decoction, Chinese patent medicine, and Chinese medicine preparation (such as tablet, pill, powder, cream, Dan, and so on) will be included. Other therapies of TCM, such as acupuncture, massage, Qigong, are not included in the study. The interventions in the control group are routine treatment of western medicine, including oxygen therapy, infection control, nutritional support, and Antifibrotic drugs (pirfenidone, nintedanib, and NAC).^[1]^

#### 2.1.4 Types of outcome measures

According to clinical investigation and research, almost 80% of IPF patients suffer from chronic cough, which is one of the important reasons for the decline of patients QOL. Cough is closely related to the time of death in patients with IPF and is an independent predictor of disease progression.^[20]^ Cough and the decline in QOL caused by cough have caused great pain to patients with IPF. For the treatment of IPF, in addition to prolonging the survival time of patients, reducing clinical symptoms and improving the QOL is an important treatment goal at present. Therefore, cough frequency and cough-related QOL are the main outcomes, while adverse reactions and safety events were secondary outcomes.

1. Cough frequency, assessed as a continuous outcome (e.g., mean change in cough, measured by cough monitoring devices or manual counting) or as a dichotomous outcome (e.g., proportion of participants reporting complete or partial relief of cough).
2. Cough-related QOL, assessed by cough questionnaires including not limited to cough visual analogue scale (VAS),^[21]^ Leicester cough questionnaire (LCQ),^[22]^ Cough Quality of Life Questionnaire (CQLQ),^[4]^ St. Georges Respiratory Questionnaire (SGRQ).^[23]^

Secondary outcomes: Adverse reactions, any adverse events outside the purpose of treatment will be considered.

### 2.2 Search methods

The following electronic databases will be systematically searched without language and time limit: English document database including Medical Literature Analysis and Retrieval System Online (MEDLINE, via PubMed), Excerpta Medica database (EMBASE, via Elsevier), Cochrane Central Register of Controlled Trials (CENTRAL), and Chinese document database including China National Knowledge Infrastructure (CNKI), VIP Chinese Science and Technology Periodical Database (VIP), China Biology Medicine (CBM), and Wanfang database. The search strategy will consist of medical subject headings and free-text terms related to “Idiopathic Pulmonary Fibrosis”, “IPF”, “Drugs, Chinese Herbal”, “Chinese herbal Medicine”, “Medicine, Chinese Traditional”, “Traditional Chinese Medicine”.

### 2.3 Selection of studies

**Figure 1:**
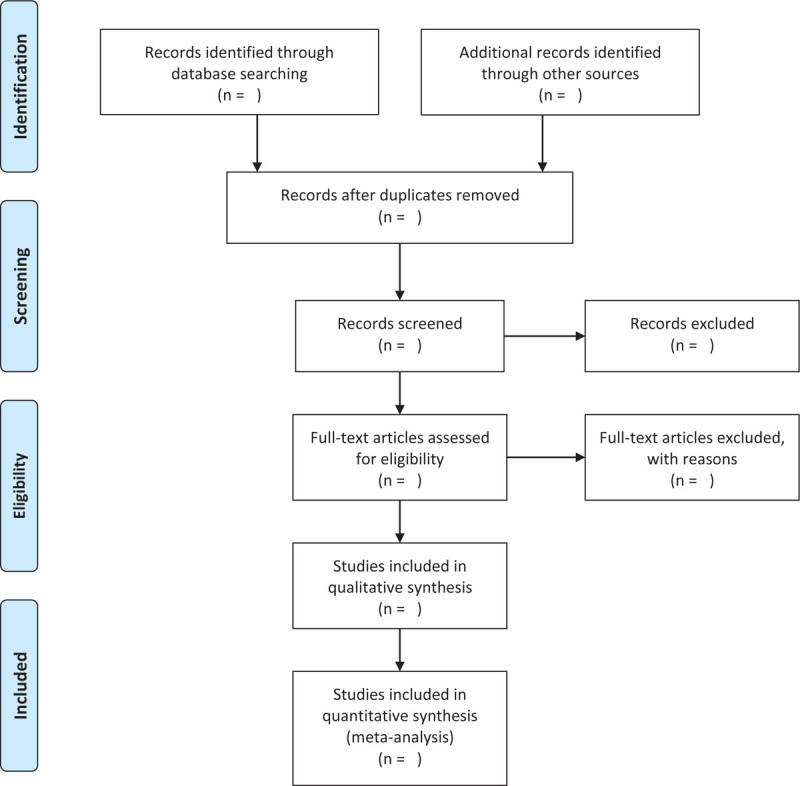
Preferred reporting items for systematic reviews and meta-analyses flow chart of study selection process.

Two independent reviewers (SY Xiao and YM Xiong) will excluding duplicates articles using NoteExpress (version:3.2; Beijing Aegean Software Company, Beijing, China) and remove irrelevant articles by reading the title and abstract, and then evaluate the trials to determine if they are appropriate for inclusion by reading the full text. Any disagreement will be resolved by discussion with the third team members (Yang Yu). Full-text articles of eligible studies will be analyzed in detail, and then reviewers will decide which studies to include and explain the reasons for excluding the study. The selection process will be recorded in detail, and the PRISMA flow diagram and tables will be filled in with the characteristics of the included and excluded studies.^[17,18]^ The whole selection process will be presented in a PRISMA flow diagram (Fig. 1).

### 2.4 Data collection and extraction

Two reviewers (SY Xiao and YM Xiong) will extract the data independently using an excel data extraction form devised by the team. The following data on the study characteristics and outcomes of the included studies will be extracted:^[19]^

1. General information: research ID, primary author, title, year of publication, country of study, and report sources.
2. Methods: study design, generation of the allocation sequence, allocation concealment, blinding, selective reporting, study sample size, the total duration of the study, number, and location of study centers.
3. Participants: number, age, gender, population, course of the disease, the severity of the condition, diagnostic criteria and inclusion/exclusion criteria, reasons, and the number of patients who dropped out or lost during follow-up.
4. Interventions: intervention, comparison, syndrome differentiation of TCM, types, and dosage form of CHMs, duration of treatment.
5. Outcomes: primary and secondary outcomes (adverse events: including main symptoms of adverse reactions, the number of patients with safety incidents), and time points reported.

One reviewer (SY Xiao) will enter the above data into Review Manager software (RevMan, Version 5.3 for windows, The Cochrane Collaboration, Oxford, England), for statistical analysis. If the results are not applicable to meta-analysis, a descriptive analysis will be carried out.

### 2.5 Risk of bias assessment

Two independent reviewers (SY Xiao and JX Yan) will use the Cochrane collaborations tool to assess the risk of bias of the included studies based on recommendations in the Cochrane Handbook.^[19]^ Risk of bias mainly includes the following: random sequence generation, allocation concealment, blinding of participants and personnel, blinding of outcome assessment, incomplete outcome data, selective reporting, and other bias. The assessment tool will classify the study as low risk, high or unclear risk of bias for each of the domains. Any disagreements will be resolved by discussion with the third reviewer (Yang Yu).

### 2.6 Data synthesis and analysis

RevMan5.3 software will be used to synthesize and analyze the data of outcomes. The studies will be assessed for heterogeneity using the *I*^2^ test. *I*^2^ values of 25%, 50%, and 75% correspond to low, moderate, and high heterogeneity, respectively.^[24]^ If more than 10 studies are included, visual inspection of the forest plots will be used to assist in the analysis of statistical heterogeneity with the corresponding 95% CI. After the *I*^2^ test and forest plots, if the included studies are considered to have acceptable heterogeneity, a meta-analysis will be conducted. Otherwise, we will consider subgroup analysis and sensitivity analysis to analyze the source of heterogeneity. According to Cochrane recommendations, population, interventions, comparators, outcomes characteristics, and *I*^2^ values will be considered to determine whether there is heterogeneity between studies.^[19]^ We will use a fixed-effects model for data analysis when it is determined that there is no heterogeneity or low level of heterogeneity does not affect the results. On the contrary, *I*^2^ > 50% or significant clinical differences regarding patients and interventions of indicating substantial heterogeneity exists in the pooled studies, the random-effects model will be adopted.

Subgroup analysis will be conducted according to the characteristics of the patients (e.g., the severity of the condition) and characteristics of the interventions (e.g., different interventions, treatment duration) to explore the possible causes of heterogeneity. Sensitivity analysis will be carried out to eliminate the impact of high-risk studies to ensure the accuracy of the results, which have a high risk of bias in at least 1 main domain in the risk of bias tool.^[19]^

### 2.7 Publication bias

The presence of publication bias and other types of reporting bias will be explored by funnel plot analysis (the number of studies included >10).^[25]^

### 2.8 Quality of evidence

This study will use the Grading of Recommendations Assessment, Development, and Evaluation (GRADE) software (GRADE pro) to evaluate the quality of evidence. The 5 GRADE criteria that may reduce the quality of evidence will be considered: limitations of the study, consistency of effect, imprecision, indirectness, and publication bias. The summary of findings table (SoF table) will be formed by using GRADE pro online based on Cochrane Handbook.^[19]^

### 2.9 Ethics and dissemination

This research is a systematic review and meta-analysis, patients will not be treated directly in the course of the study, and the personal information of patients will not be disclosed. Therefore, informed consent and ethical permission are not required for our research. We intend to update the public registry with this review in all phases of its execution and publish the results in a widely accessible journal after the completion of the research.

## 3 Discussion

The protocols reveal an explicit plan of a systematic review focusing on the efficacy and safety of CHMs in the treatment of cough in IPF. Chronic cough is one of the most common symptoms in IPF patients, and about 80% of patients have a cough to varying degrees. Cough caused by IPF is one of the main reasons for reducing the QOL of patients during the disease and related to predicting the occurrence and progression of the disease. Based on the guidelines on cough associated ILD, there is a temporary lack of effective and safe drugs to relieve cough in IPF.^[5]^ In previous meta-analyses of IPF treatment, most studies focused on the outcome index to prolong survival and delay the decline of lung function, the research on cough of IPF is blank. Compared with improving the value of lung function, relieving clinical symptoms, and improving patients QOL may be more valuable to patients with IPF.

IPF is considered to belong to the category of “Fei Wei” and “Fei Bi” in TCM. CHMs combined with conventional western medicine has been widely used in the treatment of IPF in China. Some studies have found that CHMs has obvious efficacy in relieving cough and improving the QOL of IPF patients, but there is still a lack of high-quality evidence-based evidence, resulting in the lack of reliable basis for clinical application of CHMs.^[26]^ Therefore, we drafted this review to explore the efficacy and safety of CHMs in relieving cough in IPF. Aiming at the key symptom of cough in IPF, the protocol takes indicators related to cough (such as cough frequency and QOL related to cough) as results and ensures the normal implementation of this study through scientific design, standardized methods, and reasonable personnel arrangement. In addition, we hope to objectively evaluate the clinical efficacy and safety of CHMs through this study, and also provide a reference value for high-quality clinical RCTs of CHMs in the future.

In summary, compared with previous studies, our work has the following innovations. First of all, we define the outcome index as chronic cough and cough-related changes in QOL. At a time when there is a lack of effective drugs to slow the progression of IPF patients, relieving clinical symptoms, and improving QOL may be beneficial for patients. Secondly, we choose CHMs as an intervention. CHMs have played a role in relieving symptoms in some cases, but it is still a lack of evidence-based evidence.

## Author contributions

Siyao Xiao and Yang Yu are joint first authors. Shunan Zhang, Siyao Xiao, and Yang Yu designed the study. Siyao Xiao and Yimin Xiong drafted the initial manuscript. Siyao Xiao, Xiaoyu Liu, and Jiaxin Yan designed the statistical methodology for the study. Yang Yu and Fang Sun contributed to the critical revision of the manuscript.

All authors approved the final version of the manuscript.

**Conceptualization:** Siyao Xiao, Yang Yu, Shunan Zhang.

**Funding acquisition:** Yang Yu, Shunan Zhang.

**Methodology:** Siyao Xiao, Yimin Xiong, Xiaoyu Liu, Jiaxin Yan.

**Software:** Xiaoyu Liu.

**Supervision:** Shunan Zhang.

**Writing – original draft:** Siyao Xiao, Yimin Xiong.

**Writing – review & editing:** Yang Yu, Fang Sun.
